# Discovering and identifying New York heart association classification from electronic health records

**DOI:** 10.1186/s12911-018-0625-7

**Published:** 2018-07-23

**Authors:** Rui Zhang, Sisi Ma, Liesa Shanahan, Jessica Munroe, Sarah Horn, Stuart Speedie

**Affiliations:** 1 Institute for Health Informatics, University of Minnesota, Minneapolis, MN USA; 2 College of Pharmacy, University of Minnesota, Minneapolis, MN USA; 3 Department of Medicine, University of Minnesota, Minneapolis, MN USA; 4 Medtronic, Inc., Minneapolis, MN USA

**Keywords:** Clinical notes, Electronic health records, New York heart association (NYHA), Natural language processing

## Abstract

**Background:**

Cardiac Resynchronization Therapy (CRT) is an established pacing therapy for heart failure patients. The New York Heart Association (NYHA) class is often used as a measure of a patient’s response to CRT. Identifying NYHA class for heart failure (HF) patients in an electronic health record (EHR) consistently, over time, can provide better understanding of the progression of heart failure and assessment of CRT response and effectiveness. Though NYHA is rarely stored in EHR structured data, such information is often documented in unstructured clinical notes.

**Methods:**

We accessed HF patients’ data in a local EHR system and identified potential sources of NYHA, including local diagnosis codes, procedures, and clinical notes. We further investigated and compared the performances of rule-based versus machine learning-based natural language processing (NLP) methods to identify NYHA class from clinical notes.

**Results:**

Of the 36,276 patients with a diagnosis of HF or a CRT implant, 19.2% had NYHA class mentioned at least once in their EHR. While NYHA class existed in descriptive fields association with diagnosis codes (31%) or procedure codes (2%), the richest source of NYHA class was clinical notes (95%). A total of 6174 clinical notes were matched with hospital-specific custom NYHA class diagnosis codes. Machine learning-based methods outperformed a rule-based method. The best machine-learning method was a random forest with *n*-gram features (F-measure: 93.78%).

**Conclusions:**

NYHA class is documented in different parts in EHR for HF patients and the documentation rate is lower than expected. NLP methods are a feasible way to extract NYHA class information from clinical notes.

## Background

Heart Failure (HF) is a progressive condition associated with high morbidity and mortality rates. Approximately 8% of the population in developed countries over the age of 75 [1] is affected by HF. The estimated 5.7 million Americans with heart failure account for more than 1 million hospital admissions annually [2]. Cardiac Resynchronization Therapy (CRT) is an established pacing therapy for patients with HF. Despite multiple clinical trials demonstrating the safety and efficacy of CRT, an estimated 25–30% of CRT patients do not experience clinical benefits [3–5].

Patient response to CRT is often measured using a composite score of several clinical outcomes, including mortality, heart failure hospitalization, ejection fraction (EF) measurements, and New York Heart Association (NYHA) functional classification [4–7]. NYHA class (I-IV) is a system for evaluating the severity of functional limitations from a patient’s heart failure condition (Table 1) [5]. Classification is based on the symptoms a patient experiences during activity and is most often documented in clinical dictation/notes thereby limiting computational access.

**Table 1:** Definition of NYHA classification. (adapted from [14])

NYHA Class	Patient Symptoms
I	No limitation of physical activity. Ordinary physical activity does not cause undue fatigue, palpitation, dyspnea (shortness of breath).
II	Slight limitation of physical activity. Comfortable at rest. Ordinary physical activity results in fatigue, palpitation, dyspnea (shortness of breath).
III	Marked limitation of physical activity. Comfortable at rest. Less than ordinary activity causes fatigue, palpitation, or dyspnea.
IV	Unable to carry on any physical activity without discomfort. Symptoms of heart failure at rest. If any physical activity is undertaken, discomfort increases.

Electronic health record systems are primarily designed to support the process of clinical care and billing for the services provided and contain both structured and unstructured clinical data. Some of the information such as diagnoses, procedures, or medications exists in the form of standard codes such as International Classification of Diseases, Ninth and Tenth Revision (ICD9/ICD10), Current Procedural Terminology (CPT) or RxNorm (a normalized name system for generic and branded drugs from the National Library of Medicine) which have nationally agreed upon meanings. Typically, such systems also support structured information in the form of local codes that have been created for internal use by the organization supporting the EHR. In addition to structured data, most EHRs contain clinical notes created during clinical care to document detailed patient information for billing and communication purposes. Such unstructured clinical notes are another source of valuable information that often goes well beyond the structured EHR data in characterizing patients’ medical conditions. However, as they are intended for human use rather than computational analysis, it is a considerable challenge to extract information for the purpose of secondary analyses or clinical research.

Identifying NYHA class from clinical notes in an EHR system in a systematic way could change clinical practice in several ways. Capturing NYHA class consistently over time may inform a better understanding of the progression of HF to assess CRT effectiveness and its benefit-risk profile versus other therapies and how to improve the response rate and effectiveness of CRT. A patient’s NYHA class assessment over time provides the potential to better understand why some patients benefit from CRT while others do not [3–5]. Additionally, if NYHA class can be reliably derived from other EHR values, it may provide more consistent and objective measure of an HF patient’s functional status. In our prior study [8], we have demonstrated the feasibility of leveraging NLP techniques to extract NYHA class from clinical notes.

To the best of our knowledge, there has been no study investigating the sources of NYHA class in the EHRs and assessing the data availability for NYHA class pre- and post-implant of CRT devices. We further improved the performance of NLP algorithms, comparing with our prior work, to extract specific outcome data (NYHA class) for patients with a CRT device from electronic health records. This study explores the possibility of monitoring CRT effectiveness as measured by NYHA class more efficiently and potentially improve health care for HF patients.

## Methods

### Data sources

The patient population for this study was drawn from clinical data documented between December 1, 2011 and January 1, 2016 by Fairview Health Services (FHS). Data containing approximately 2.6 million patients extracted from FHS’s EHR resides in a Clinical Data Repository (CDR) maintained by the University of Minnesota Academic Health Center.

### Study overview

**Fig. 1:**
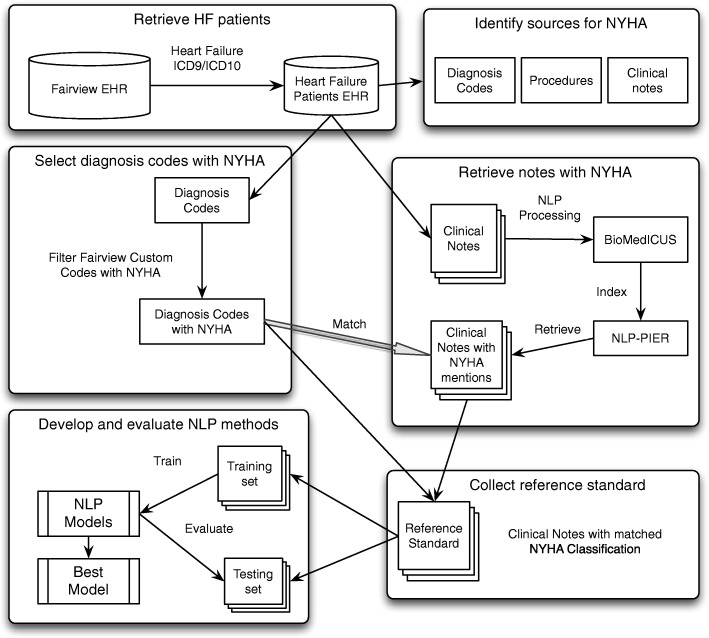
Overview of methodology

As shown in Fig. 1, we undertook the following steps to execute this study: 1) retrieved EHR data for heart failure patients; 2) identified sources of NYHA classifications; 3) selected local diagnosis codes that corresponded to an NYHA classification; 4) retrieved clinical notes with explicit mentions of NYHA; 5) collected a subset of clinical notes as reference standard; 6) developed and evaluated NLP methods. We will describe each step, in detail below.

### Retrieving EHR for heart failure patients

In this study, we focused on a heart failure patient cohort identified using diagnosis codes associated with a heart failure. All patients who had at least one diagnosis of heart failure (as defined by the list of International Classification of Diseases, Ninth and Tenth Revisions (i.e., ICD9 and ICD10 codes) in Table 2 were included. Associated with each encounter were age at encounter, ICD9 or ICD10 encoded diagnoses, local coding that informed the standard diagnostic codes, procedures with associated narrative comments, and clinical notes. The resultant information was organized into a datamart of related tables within the secure environment that were employed to carry out the research activities for the above objectives. All start dates were approximate and depended on the date for the adoption of the EHR at each particular site.

**Table 2:** A list of diagnosis codes for identifying health failure patients

ICD Revisions	ICD codes
ICD9	398. 91, 428. *, 402. 01, 402. 11, 402. 91, 404. 01, 404. 03, 404. 11, 404. 13, 404. 91, 404. 93, 428. 1–428. 4, 428. 9
ICD10	I11. 0, I13. 0, I13. 2, I50. 1-I50. 4, I50. 9, I97. 13, I50. 4, I50. 9

### Identifying sources of NYHA classifications

The first step in the process was to investigate the possible sources of NYHA Class within the datamart. This involved a systematic search for explicit mentions of NYHA Class in the custom diagnostic codes’ descriptive names, both local and standard, as well as in all the descriptive information (such as “Narratives”) associated with procedures and observations. Any mention of NYHA class or its synonyms (e.g., New York Heart Association, etc.) was noted. The availabilities of NYHA class in the various EHR data sources were compared. All sources of NYHA class were identified and characterized in terms of frequency of occurrence. Furthermore, since NYHA Class is often used as a clinical outcome measure for CRT patients, its availability for these patients over a five-year timespan was also examined. The subset of patients who had a CRT implant and at least one NYHA Class was used for the purpose of developing a model for estimating NYHA Class for a patient encounter from structured data available from the EHR for that encounter.

### Selecting diagnosis codes with NYHA classification

To collect NYHA class labels, we selected patients having encounters associated with Fairview custom diagnosis codes with NYHA information. Such Fairview custom codes, with labels like “Congestive heart failure with left ventricular diastolic dysfunction, NYHA class 3 (H)” indicated a NYHA classification III in this case. These local custom code strings with NYHA information were extracted and mapped to the corresponding NYHA classification (I – IV) based on the statements in the code names.

### Retrieving clinical notes with NYHA mentions

**Fig. 2:**
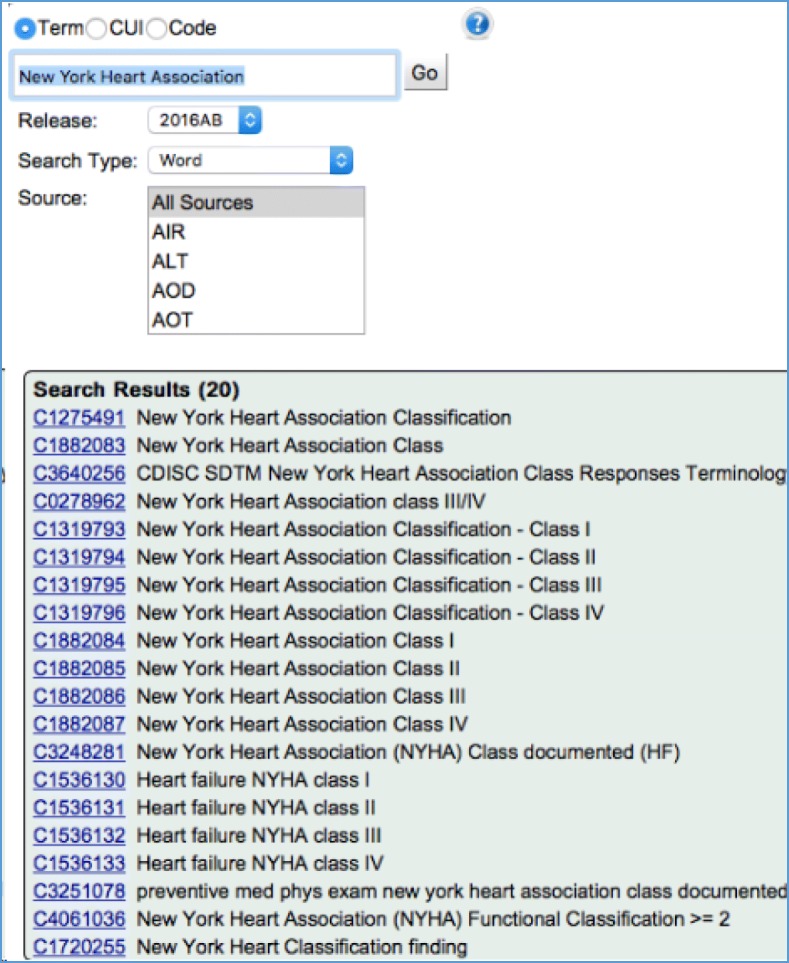
UMLS CUIs associated with NYHA classification used for retrieving clinical notes

To collect the corpus of text notes for this study, the NLP web-based search engine, Patient Information Extraction from Researchers (PIER) [9] was used to search clinical notes containing NYHA concepts. Before the searching, clinical notes in the CDR were first processed by BioMedICUS (BioMedical Information Collection and Understanding System) [10], which is an NLP system based on the Unstructured Information Management Architecture – Asynchronous Scaleout (UIMA-AS) architecture. BioMedICUS identifies Unified Medical Language System (UMLS) Metathesaurus concepts associated with a note that are stored in an Elasticsearch cluster along with the source note itself. This allows PIER to search notes through UMLS concept unique identifiers (CUIs) or keywords for NYHA. To retrieve clinical notes with NYHA mentions, we searched for the CUIs listed in Fig. 2 that were obtained using the UMLS Metathesarus browser. We also conducted keyword searching using “New York heart association”, “NYHA”, “New York heart classification”, “NY classification”, “NY class” to cover the lexical variations for NYHA.

### Developing a reference standard

We refer the reference standard as the clinical notes with their associated NYHA classes. To create such a NYHA reference standard to train and evaluate the NLP algorithms, we matched the clinical notes that were documented to encounters with at least one Fairview NYHA Class diagnosis code (where NYHA class can be determined) for the same patient and same date. It is assumed that the notes should mention the same NYHA classification as associated NYHA Fairview diagnosis code within a single patient encounter. We then assigned the NYHA class associated with Fairview custom codes to the clinical notes in the same encounter. The notes with the assigned NYHA as labels were selected as a reference standard for developing and evaluating our NLP methods. A total of 6174 clinical notes with an associated NYHA classification were obtained.

### Developing NLP methods to identify NYHA classes in clinical notes

To select the best method, we evaluated and compared a rule-based method with machine learning-based methods to extract NYHA class from clinical notes. We split the corpus into 3700 notes as a training data set and 2474 notes as a testing set.

#### Rule-based method

Rules were created by iteratively reviewing the notes to discover patterns. The training set was used to refine the patterns to capture most expressions for NYHA classes. Co-occurring NYHA classes such as “NYHA II/III” were also recognized. Listed below are summarizations of the patterns to identify classifications that appeared within 40 characters after a NYHA string such as “NY class”:

- Single digit 1, 2, 3, and 4;
- Single Roman numeral I, II, III, and IV;
- Two digits or Roman numerals (between range 1 to 4, inclusive), separated by single character ‘-’ or ‘/’. Space can be inserted/omitted between characters. Only patterns related to two contingency classes, such as NYHA 1–2 and NYHA II–III, were considered.

#### Machine learning-based methods

Clinical notes were pre-processed and features were generated. Details in each step are described below:

- The classic stopwords [11] (such as “of”, “as”) were removed from the texts. This step deemphasizes these less important words for building the model.
- To normalize lexically different forms of the same term as equivalent, lexical variant generation (LVG) was used.
- The words based on their term frequency in the corpus were ranked. The words within the lowest 1% of the rank were discarded. This step removes the least frequently used words from the model development process.
- Feature set 1 includes “bag-of-words” features.
- Feature set 2 includes *n*-gram features where *n* was from 2 to 5.
- Three widely used machine learning algorithms (Support Vector Machine, Logistical Regression, and Random Forest) were applied with both feature sets to determine NYHA classification for each encounter, and compared in the training corpus using 10-fold cross validation.

#### Evaluation

Both rule-based and machine learning-based methods were applied to the same held-out testing dataset and generated the most likely NYHA classification for each encounter based on the features in the associated set of clinical notes. The resulting NYHA classes were compared with the NYHA classes from the reference standard corpus. Evaluation metrics, including precision, recall, and F-measure, were used for each class and overall NYHA class assignment for three machine learning methods with each of the two features sets.

## Results

### Identifying sources of NYHA classifications

Examination of the datamart data elements revealed that there was one structured data item that contained NYHA class information. That was the local diagnosis code that was generated in the system and used to determine the standard ICD9/ICD10 diagnosis code. There was a total of 260 such codes associated with ICD9/ICD10 codes for HF. A typical code description was “CHF (NYHA class III, ACC/AHA stage C) (H)”. A second source of NYHA Class information was in the Impression/Narrative field associated with procedures. These fields were examined with a straightforward string searching approach to identify NYHA information. No NYHA Class information was found in the table of observations. The most productive source of information about NYHA class was the clinical note.

**Fig. 3:**
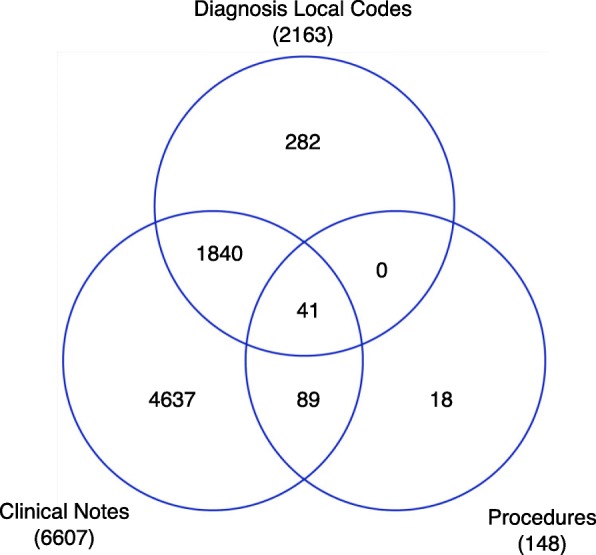
Overlap of number of patients for various NYHA classification sources in the EHR

We initially identified 36,276 patients that met the inclusion criteria (a diagnosis of HF or evidence of a CRT device). 35,900 of those had a HF diagnosis and 376 had evidence of a CRT without a HF diagnosis. Descriptive statistics for various patient cohorts was summarized in Table 3. In addition, The NYHA Class sources and frequencies results per patient are summarized in Fig. 3. Among all three resources, the richest information was from clinical notes (95.6%) as opposed to diagnosis local codes (31.3%) and narrative filed with procedures (2.1%). There are few patients (0.6%) who can be identified from all three resources. NYHA class was available for 6907 patients with a HF diagnosis, i.e., 19.2% of all HF patients. Those with a CRT device had a 72.9% rate of NYHA documentation per patient.

**Table 3:** Statisics for various patient cohorts

Cohort definition	Number of patients
Patients who have a diagnosis of HF or evidence of a CRT device	32,276
Patinets who have a HF diagnosis	35,900
Patients who have evidence of a CRT device without a HF diagnosis	376
Patients with a HF diagnois having NYHA mentions	6907
Patients who have evicence of a CRT device (with or without a HF diagnosis) and NYHA mentions	696
Patients who have NYHA mentions in clinical notes and corresponding local diagnosis codes	1370

The second step was to examine the documentation of an NYHA Class over time for a group of patients who had received a CRT implant. The group consisted of 696 patients with 51,138 encounters over a five-year period post-implant. Table 4 summarized the frequency of this documentation before and after implantation. The percentage of patients with at least one instance of a documented NYHA class ranged from 51.6% for the pre-implant period to 30.0% for year 5 post implant. The proportion of encounters with an associated NYHA Class remained fairly similar over the post-implant years (range: 16.7 to 25.5%). As expected both the number of CRT patients and their instances of NYHA Classification declined over time likely due to both the patient survival rate and to movement to other health care organizations. The observed lower rate of NYHA Class per encounter in the pre-implant period was quite possibly due to the larger number of encounters during that period.

**Table 4:** NYHA documentation over time for CRT patients

	Pre Implant	Post Implant				
		Year 1	Year 2	Year 3	Year 4	Year 5
CRT Patients	696	600	485	405	310	212
CRT Patients with NYHA Class	51.6%	35.7%	27.4%	25.3%	24.6%	30.0%
Encounters	11,844	5550	3984	3171	2174	1740
Encounters with NYHA Class	10.5%	25.5%	18.3%	16.7%	19.9%	20.7%

### Evaluating NLP methods to identify NYHA classification from clinical notes

A total of 6174 clinical notes from 6039 encounters for 1370 patients was obtained as reference standard. Table 5 lists the distribution of NYHA classes in the entire corpus as well as the training and testing sets. Overall, NYHA class II had the largest proportion (22%) and class IV the smallest (8%).

**Table 5:** Number of clinical notes in training and testing set

NYHA Classification	Training Set	Testing Set	Total
I	843	524	1367
II	1506	996	2502
III	1045	745	1790
IV	306	209	515
Total	3700	2474	6174

**Fig. 4:**
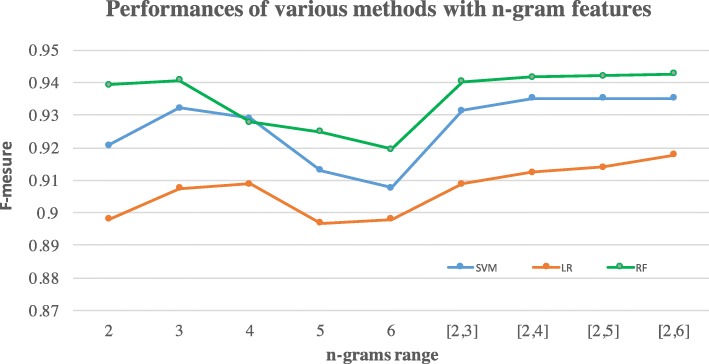
Performance comparison of machine learning methods with various n-gram ranges. Notes: [2, 5] indicates the n-grams feature where range for *n* is from 2 to 5

The rule-based method performed well overall (F-measure: 93.54%), with its performance varying from the lowest of 86.16% for Class IV to the highest of 96.41% for Class II (See Table 6). The best machine learning-based method random forest with *n*-gram features (overall F-measure: 93.78%) slightly outperformed the rule-based method and significantly outperformed those with bag-of-word features (Table 7). The performance is slightly lower than its performance (F-measure: 96.0%) in the training set using 10-fold cross validation. This is also a better performance compared with our prior work [8]. We also compared the various n-grams ranges and found the best performance with the features from bigram to five-grams (Fig. 4). On both feature sets, the random forest method performed the best among three machine learning algorithms. The F-measures for extracting NYHA class I, II and III are over 92% with respect to the reference standard for both rule-based and machine learning-based methods with *n*-gram features. Table 8 lists the top bag-of-words and *n*-gram features for those methods. For all methods, they were most accurate in identifying Class II and least in identifying Class IV.

**Table 6:** Performance of rule-based method

NYHA Classification	Precision	Recall	F-Measure
I	95.07%	97.39%	96.21%
II	95.72%	97.10%	96.41%
III	94.34%	95.07%	94.70%
IV	94.83%	78.95%	86.16%
Overall	94.99%	92.13%	*93.54%*

Italics indicate the best performance

**Table 7:** Performances of machine learning-based methods

NYHA Classification	Precision	Recall	F-Measure
Feature Set 1: bag-of-words			
Support Vector Machine			
I	84.71%	82.06%	83.36%
II	88.30%	91.80%	90.01%
III	88.34%	88.46%	88.40%
IV	80.00%	70.81%	75.13%
Overall	85.34%	83.28%	84.23%
Logistic Regression			
I	83.61%	79.97%	81.75%
II	86.87%	91.99%	89.36%
III	87.66%	88.46%	88.06%
IV	80.72%	64.11%	71.47%
Overall	84.72%	81.13%	82.66%
Random Forest			
I	87.54%	86.93%	87.24%
II	91.23%	94.40%	92.79%
III	91.83%	90.40%	91.11%
IV	79.89%	72.25%	75.88%
Overall	87.63%	86.00%	86.75%
Feature Set 2: n-gram			
Support Vector Machine			
I	95.05%	93.73%	94.39%
II	95.71%	96.81%	96.26%
III	94.95%	95.20%	95.08%
IV	89.66%	87.08%	88.35%
Overall	93.84%	93.21%	93.52%
Logistic Regression			
I	93.09%	91.46%	92.27%
II	94.74%	95.66%	95.20%
III	93.12%	94.81%	93.96%
IV	87.18%	81.34%	84.16%
Overall	90.03%	90.82%	90.42%
Random Forest			
I	97.02%	96.52%	96.77%
II	97.58%	97.49%	97.54%
III	93.01%	96.63%	94.78%
IV	93.99%	82.30%	87.76%
Overall	95.40%	92.23%	93.78%

**Table 8:** Top 15 n-gram features from feature sets 1 and 2

Rank	Feature set 1: Bag of words	Feature set 2: n-grams
1	ii	nyha class ii
2	iii	class ii
3	iv	nyha class iii
4	b	class iii
5	428.0dd	nyha class iv
6	428.0db	class iv
7	chf	nyha class ii
8	congestive	nyha class 2
9	428.0 dc	nyha class 3
10	lvad	class
11	systolic	chf
12	diastolic	class 3
13	428.0bm	class 2
14	c	congestive heart failure
15	stage	nyha class

## Discussion

NYHA class for HF patients proved to be rather elusive. It was documented in heterogeneous types of data, including diagnostic local codes, clinical notes and the description component of procedures, none of which comprehensively documented NYHA class. Clinical notes appeared to be the best source for NYHA data in the EHR though local diagnostic information identified approximately 27% more patients with NYHA documentation. Extraction of NYHA class from unstructured notes required significant NLP data analysis and we demonstrate the feasibility and accuracy of using such methods to automatically identify existing NYHA class in those notes.

We conclude that it is possible to retrieve a significant amount of NYHA class information for HF patients from the EHR using a combination of clinical notes and local diagnosis codes. The use of local diagnosis codes is somewhat problematic as it is not a particularly generalizable approach across EHRs. However, the extent to which the overlap between clinical notes and the local diagnostic codes is driven by the coding process of Health Information Management coding specialists may serve as a guidepost to further extraction of NYHA Class from notes.

The overall rate of NYHA Class documentation for HF patients was slightly less than 1 in five (19%). The American College of Cardiology recommends that “NYHA Class should occur at each office visit to quantify the degree of functional limitation imposed by HF” [12]. It is obvious that the documentation rate falls far short of this recommendation for the population of HF patients in this study. While there are numerous possible reasons why NYHA Class might be missing including patient attrition from the health system and documentation in locations other than the EHR, it is certainly possible that providers are not yet consistently following this recommendation. Further investigation of this observation is required.

As was expected, NYHA class was documented with a much higher frequency in CRT patients. Almost four out of every five (79.2%) of these patients had at least one instance of an NYHA class recorded. Examination of the pre-implant period when it was available indicated that about half of the patients (51.9%) had an NYHA class documented prior to the date of implant. Through manual inspection we observed that this did not always occur during the encounter in which the implant was performed. This higher rate is to be expected as a NYHA class is one of the factors used to determine the need for CRT as well as a measure of effectiveness once the implant takes place. Yet the rate still falls short of the expected 100% for these patients. While there are a range of explanations similar to those for the general HF population, further investigation is required.

The more detailed examination of the rate of NYHA Class documentation over time revealed that it remained fairly constant over the five-year time span post-implant at a rate of approximately 16–25% of annual patient encounters. Since these encounters were limited to those identified as “face-to-face” encounters with clinicians, it is reasonable to assume that they would provide opportunities for documenting NYHA class. We did observe an expected decline in the numbers of CRT patients with encounters over the five-year period certainly due in part to the survival rate of CRT patients which has been reported as a median of 6–8 years [13], but also quite possibly due to patients leaving the health system for a variety of non-clinical reasons. It is important to note that we did not distinguish between encounters with cardiologists and other clinical specialties including primary care. It is possible and reasonable that the documentation rate was higher for cardiology encounters than for other specialists which would at least partially account for the lower than expected overall rate for encounters.

Applying NLP algorithms to those associated notes indicated that for over 95% of the encounters where NYHA was mentioned in the clinical notes, it matched the custom code NYHA class. This is to be expected given that these local diagnosis codes were assigned by experts after they reviewed clinical notes. Similar to the rule-based method, machine learning-based methods performed well on NYHA class I-III. Performance of machine learning-based algorithms with *n*-grams performed better, due to the fact that bag-of-word features miss the ordering information of words like *n*-grams. With such context information, the performance generally improved about 8%. The smaller proportion (only 8%) of NYHA class IV mentions, compared with other NYHA classes that were better represented in the dataset, may have led to a lower performance on both NLP methods for this class. Further investigation is needed to identify other possible reasons for the low recall for class IV.

After carefully reviewing some wrongly identified instances, we found two categories of error. One was from the cases where the NYHA class was ambiguous, such as “NYHA 1 or 2”. The rules only allowed the contiguous classes with the AND logic link, such as “NYHA 1–2”. Some NYHA mentions were not a diagnosis statement, but from the section of recommendation or history. Our method currently cannot differentiate between historical NYHA class and current NYHA class in the notes. Our reference standard is built from the local diagnosis codes on the same encounter, which usually reflect the current NYHA classes during the encounter.

There are limitations which need further investigation. We focused solely on a matched set of notes and NYHA local codes for the same patients. The generalizability of algorithms across the larger and more varied body of clinical notes available in a typical EHR will need further validation, although the machine learning models with *n*-gram features perform exceptionally well. Further work employing NLP techniques with notes in which NYHA class is not explicitly identified may well lead to a higher rate of identification based on other relevant data in those notes. Information relating to NYHA class was also found in various text-based comment fields throughout the record. Future work would include those text fields to explore their value in further improving the performance of the NLP model.

## Conclusions

NYHA classification is not well-documented in structured data but rich NYHA information is stored in unstructured clinical notes. We developed a rule-based method and compared it to machine learning methods to identify NYHA class I-IV from those notes. The Random Forest method with n-gram features performed best in identifying NYHA I-III Class. Further validation of these results and methods is required.

## Abbreviations

CDR: clinical data repository
CRT: Cardiac Resynchronization Therapy
EHR: electronic health records
FHS: Fairview Health Services
HF: heart failure
NLP: natural language processing
NYHA: New York heart association
